# The interplay between ATF2 and NEAT1 contributes to lung adenocarcinoma progression

**DOI:** 10.1186/s12935-020-01697-8

**Published:** 2020-12-09

**Authors:** Jian Liu, Kai Li, Rui Wang, Sisi Chen, Jie Wu, Xiang Li, Qian Ning, Ganghua Yang, Yamei Pang

**Affiliations:** 1 Department of Respiratory and Critical Care Medicine, the First Affiliated Hospital of Xi’an Jiaotong University, 277 Yanta West Road, Xian, 710061 Shaanxi China; 2 Department of Thoracic Surgery, the First Affiliated Hospital of Xi’an Jiaotong University, Xi’an, 710061 Shaanxi China; 3 Department of Geriatric Surgery, the First Affiliated Hospital of Xi’an Jiaotong University, 277 Yanta West Road, Xian, Shaanxi 710061 People’s Republic of China

**Keywords:** ATF2, NEAT1, miR-26a-5p, Lung adenocarcinoma

## Abstract

**Background:**

Activating transcription factor 2 (ATF2), a member of the activator protein 1 (AP-1) transcription factor family, has been shown to be involved in the pathobiology of numerous cancers. However, the biological role and mechanism of ATF2 in lung adenocarcinoma (LUAD) remains to be elucidated.

**Methods:**

The expression of ATF2, NEAT1 and miR-26a-5p in LUAD tissues and cell lines was detected by qRT-PCR and western blotting. The interaction between ATF2, NEAT1, and miR-26a-5p was validated by chromatin immunoprecipitation, luciferase reporter assay and RNA immunoprecipitation. Cell proliferation, invasion and tumorigenesis of LUAD cells were analyzed by using CCK8, transwell invasion assay and xenograft tumor model.

**Results:**

We confirmed that ATF2 expression was increased in LUAD tissues compared with normal adjacent lung tissues. Functional experiments showed that ATF2 positively regulated cell proliferation and invasion in LUAD cells. Moreover, we identified that NEAT1 expression was increased in LUAD tissues and positively correlated with ATF2 expression. Mechanistically, ATF2 could bind to the promoter of NEAT1 to promote its transcription. Rescue experiments showed that ATF2 exerted its oncogenic function in LUAD, at least, partly through NEAT1 upregulation. In turn, NEAT1 could positively regulate ATF2 expression and form a positive feedback loop in LUAD cells. Furthermore, we demonstrated that NEAT1 positively regulated ATF2 expression via sponging miR-26a-5p.

**Conclusion:**

ATF2 and NEAT1 form a positive feedback loop mediated by miR-26a-5p and coordinately contribute to LUAD progression.

## Background

Lung cancer is one of the most prevalent malignant tumors and the leading cause of cancer related mortality worldwide [1]. Lung adenocarcinoma (LUAD), a major histological subtype of lung cancer, accounts for 40% of lung cancer cases and leads to 500,000 cancer related death every year [2]. Although great progress has been made in the diagnosis and treatment in the past years, the average five-year survival rate of LUAD patients remains less than 20% due to tumor heterogeneity and aggressiveness [3]. In order to improve the clinical outcomes of LUAD patients, a deep-going investigation on the molecular mechanism underlying LUAD progression is urgently required.

Activating transcription factor 2 (ATF2) is a member of the activator protein 1 (AP-1) transcription factor family, characterized by a basic structural region and a leucine zipper domain. ATF2 is activated by JNK, p38 (MAPK14) and ERK1 through Thr69 and Thr71-phosphorylation, then translocates into the nucleus and thus activates target genes transcription [4]. The activation of ATF2 promotes the expression of a series of target genes, which refer to cell cycle, immune and inflammatory responses and apoptosis regulation [5]. However, phosphorylated ATF2 at Thr52 by PKCε binds to the IFNβ1 promoter and represses gene transcription, indicating an transcription suppressor role of ATF2 [6]. Previous studies have reported that ATF2 is involved in the pathobiology of numerous cancers. For example, ATF2 exhibits oncogenic functions in prostate cancer [7], melanoma [6, 8], renal cell carcinoma [9] and pancreatic cancer [10]. Conversely, ATF2 exerts a tumor suppressor role in breast cancer [11] and skin cancer [12], suggesting a tumor-specific characteristic of ATF2 function. Although ATF2 overexpression in non-small cell lung cancer (NSCLC) has been observed [13, 14], the biological role and mechanism of this transcription factor in LUAD remains to be elucidated.

Long non-coding RNA (LncRNA) is a type of ncRNA which is longer than 200 nt in length and does not encode any protein [15]. Nuclear Enriched Abundant Transcript 1 (NEAT1) gene encodes two transcriptional variants, namely, NEAT1_1 (3.7 kb) and NEAT1_2 [23 Kb]. Dysregulation of NEAT1 is associated with tumor growth, recurrence, metastasis and patient survival in numerous cancers [16]. LncRNAs and messenger RNAs (mRNAs) act as competing endogenous RNAs (ceRNAs)—they co-regulate each other by competing for binding to shared microRNAs (miRNAs), a family of small non-coding RNAs that are important post-transcriptional regulators [17]. NEAT1 functions as a ceRNA to promote the expression of target genes via sponging multiple tumor-suppressive miRNAs [18]. Besides, NEAT1 epigenetically modulates target genes expression via interacting with DNMT1 or EZH2 [18]. Functionally, elevated NEAT1 expression is associated with cell proliferation, migration, invasion, cancer stem cell property and chemotherapy resistance in lung cancer [19–22].

This study aimed to explore the interplay between ATF2 and NEAT1 in LUAD. In this study, we found that ATF2 expression is elevated in LUAD tissues and positively correlates with NEAT1 expression. Furthermore, we demonstrated that ATF2 promotes LUAD cells malignancy behaviors via activating NEAT1 transcription. In turn, NEAT1 positively regulates ATF2 expression via sponging miR-26a-5p, and thereby forms a feedback loop in LUAD.

## Methods

### Cell culture and tissue samples

Human lung cancer cell lines A549, H460, H1650, H1975, H1299, and a human bronchial epithelial cell line NHBE were obtained from the America Type Culture Collection (ATCC; Manassas, VA, USA). The lung cancer cell lines were cultured in Dulbecco minimal essential medium (DMEM, HyClone, Logan, Utah, USA) supplemented with 10% fetal bovine serum (FBS, Gibco, Invitrogen, Carlsbad, CA, USA), L-Glutamine (Gibco) and antibiotics (100 U/mL penicillin and 100 μg/mL streptomycin) in a humidified atmosphere of 5% CO_2_ at 37 °C. NHBE cells were cultured in RPMI-1640 medium containing low concentration of L-Glutamine (Gibco).

All clinical samples were obtained from the First Affiliated Hospital of Xi’an Jiaotong University. All participants had signed written informed consent. This study was approved by the Ethics Committee of the First Affiliated Hospital of Xi’an Jiaotong University.

### Plasmids construction

The full-length sequences of NEAT1 and ATF2 were subcloned into the pcDNA3.1 vector (Invitrogen) to construct the NEAT1-overexpressing and ATF2-overexpressing plasmids, respectively. The shRNA-insensitive mutant ATF2 plasmid was generated by site-specific mutagenesis based on the ATF2-overexpressing plasmid. The fragments containing the wild-type or mutant miR-26a-5p-binding sites of NEAT1 and ATF2 3′UTR were subcloned into the pmirGLO vector (Promega, Madison, WI, USA) to construct the luciferase reporter vectors (NEAT1-wt, NEAT1-mut and ATF2-wt, ATF2-mut). Similarly, the NEAT1 promoter fragments containing putative ATF2-binding sites were inserted into the pGL3-basic vector (Promega) for the promoter activity assay.

### Cell transfection

MiR-26a-5p mimics, miR-26a-5p inhibitors, NEAT1 small interfering RNA (siRNA), ATF2 short hairpin RNA (shRNA) vectors and the corresponding negative control were all obtained from GenePharma (Shanghai, China). The information of the oligonucleotides is provided in the Additional file 1: Table S1. The transfection of the vectors and oligonucleotides was performed using Lipofectamine 2000 (Invitrogen) according to the manufacturer's protocol.

### Quantitative Realtime PCR (QRT-PCR)

Total RNA was extracted using Trizol reagent (Beyotime, Shanghai, China) according to the manufacturer’s protocol. Reverse transcription was performed using PrimeScript™ RT Reagent kit (Takara, Dalian, China) as previously described [23]. Realtime PCR was performed using SYBR® Premix Ex Taq™ II (Takara) on a Bio-Rad CFX96 real-time PCR detection system (Bio-Rad, Hercules, CA, USA). ACTB and U6 were taken as internal controls for mRNAs and miRNAs, respectively. The relative expression levels were evaluated using the 2^−ΔΔCt^ method. The primers used in this study are provided in the Additional file 2: Table S2.

### Western blot

Total protein was extracted using ice-cold RIPA lysis buffer (Beyotime). An aliquot of denatured protein (20 μg) from each sample was subjected to 10% SDS‑PAGE gel electrophoresis and then transferred to PVDF membrane. The membrane was blocked in 5% nonfat milk for 2 h and incubated with primary antibodies against ATF2 (ab32160, Abcam, Cambriambridge, MA, USA), p-ATF2 (sc-8398, Santa Cruz Biotechnology, Santa Cruz, CA, USA) and GAPDH (10494–1-AP, Proteintech, Wuhan, China) at 4˚C overnight. The protein bands were visualized using an enhanced chemiluminescence kit (EMD Millipore, Billerica, MA, USA) after incubation with secondary antibodies.

### Cell viability assay

Cell viability was detected using Cell Counting Kit 8 (CCK8; Beyotime) according to the manufacturer’s protocol. A549 and H1299 cells were seeded in 96-well plates at a density of 5 × 10^3^ cells/well. At indicated time points (24, 48, 72, and 96 h), 10 μL of CCK8 solution was added to each well and incubated for 2 h at 37 °C. The absorbance at 450 nm was measured using a microplate reader (Bio-Rad).

### Colony formation assay

LUAD cells were seeded into 6-well plates at a density of 500 cells/well and cultured for two weeks. Culture medium was replaced every 3 days. Then the cells were fixed with methanol and stained with crystal violet. Colonies containing more than 50 cells were counted using a light microscope.

### Cell invasion assay

Cell invasion assay was performed using 24-well plates and transwell chambers precoated with Matrigel (8 μm pore size; Corning, NY, USA). LUAD cells were seeded into the upper compartment containing serum-free culture medium at a density of 1 × 10^5^ cells/well. The lower compartment was filled with 400 μL DMEM supplemented with 10% FBS. After incubation for 48 h, the non-invading cells were removed from the upper surface of the membrane. Cells in the lower chamber were fixed with paraformaldehyde and stained with crystal violet. Then the cells were imaged and counted in five random fields using a light microscope.

### Luciferase activity assay

LUAD cells (1 × 10^5^) were plated in 24-well plates overnight and co-transfected with miR-26a-5p mimics/inhibitors, ATF2 shRNA/overexpressing plasmid or corresponding negative control, and wild-type or mutant luciferase reporter vectors using Lipofectamine 2000 reagent. After transfection for 48 h, firefly and *Renilla* luciferase activities of the cell lysates were measured by using the Dual-Luciferase Reporter Assay System (Promega).

### Chromatin immunoprecipitation (ChIP)

ChIP assay was carried out in LUAD cells with the EpiQuik™ Chromatin Immunoprecipitation Kit (P-2002; Epigentek, Farmingdale, NY, USA) according to the manufacturer's instructions. The antibodies used for ChIP assay were anti-ATF2 antibody (ab32160, Abcam) and normal rabbit IgG (#2729, Cell Signaling Technology, Danvers, MA, USA). The primer for PCR was listed in the Additional file 2: Table S2.

### RNA immunoprecipitation (RIP)

RIP assay was performed using the Magna RIP RNA Binding Protein Immunoprecipitation Kit (EMD Millipore). LUAD cells were harvested and lysed using the RIP lysis buffer. Then cell lysate was incubated with RIP buffer containing magnetic beads conjugated with an anti-AGO2 antibody (#2897, Cell Signaling Technology) or normal rabbit IgG (#2729, Cell Signaling Technology) according to the manufacturer’s instructions. The coprecipitated RNA was purified and subjected to qRT-PCR to determine the levels of NEAT1, miR-26a-5p and ATF2.

### Xenograft assay

BALB/c nude mice, aged 5–6 weeks, were purchased from SLAC Laboratory Animal Co., Ltd. (Shanghai, China). A549 cells (3 × 10^6^) stably transfected with shATF2 or scramble lentivirus were suspended in 100 μL PBS and subcutaneously inoculated into the flank of the mice. Each group contained 5 nude mice. The tumor size was measured every four days. The tumor volume was calculated using the formula: V = (length × width^2^)/2. After 28 days, the mice were euthanized, and the tumors were weighed. The animal experiments were approved by the Experimental Animal Committee of Xian Jiaotong University.

### Online data acquisition

The RNA-seq FPKM data of LUAD, containing 533 LUAD tissues and 59 normal tissues, were downloaded from The Cancer Genome Atlas (TCGA) database [24]. The GSE48414 dataset [25], containing 154 LUAD tissues and 20 normal tissues, was downloaded from the Gene Expression Omnibus (GEO) database.

### Statistical analysis

Statistical analysis was conducted with SPSS 20.0 software. Unless otherwise indicated, quantitative results are presented as the mean ± standard deviation (SD) from at least three independent experiments. Student’s *t* test and one‐way analysis of variance were utilized to assess the differences between two groups and among more than two groups, respectively. Kaplan–Meier plots and log-rank tests were used for the survival analysis. *P* < 0.05 was considered statistically significant.

## Results

### ATF2 expression is elevated in LUAD

**Fig. 1:**
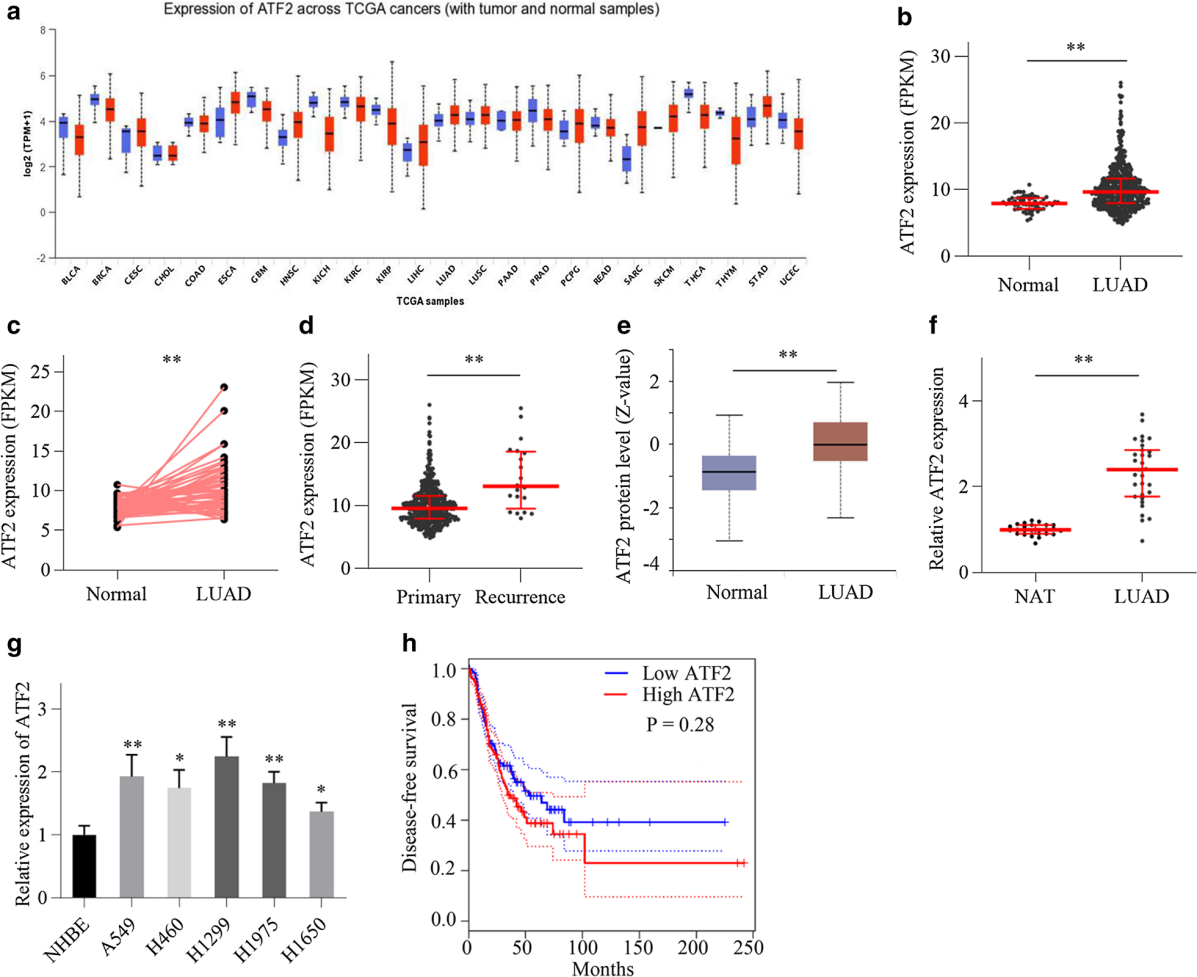
ATF2 expression is elevated in LUAD. **a** ATF2 expression in various cancers and corresponding normal tissues from the GEPIA database. Red column, cancer tissues; blue column, normal tissues. **b** ATF2 expression in LUAD tissues and normal lung tissues from TCGA database. Normal, n = 59, LUAD, n = 533. **c** ATF2 expression in paired LUAD tissues and normal lung tissues from TCGA database. Normal, n = 57, LUAD, n = 57. **d** ATF2 expression in primary and recurrent LUAD tissues from TCGA database. Primary, n = 513, Recurrence, n = 20. **e** ATF2 protein expression in LUAD and normal lung tissues based on CPTAC data from ULCAN cancer database. Normal, n = 111, LUAD, n = 111. **f** ATF2 expression in LUAD and normal lung tissues was detected by qRT-PCR. Normal adjacent lung tissues (NAT), n = 25, LUAD, n = 31. **g** ATF2 expression in lung cancer cell lines and normal bronchial epithelial cell line was detected by qRT-PCR. **h** Kaplan–Meier analysis for investigating the correlation of ATF2 expression with disease-free survival (DFS) in LUAD patients from GEPIA database. Low ATF2, n = 239, High ATF2, n = 239. ^*^P < 0.05, ^**^P < 0.01

Initially, bioinformatics analysis using GEPIA algorithm showed that ATF2 was highly expressed in several cancer types (Fig. 1a). Then we analyzed ATF2 expression in LUAD by using RNA-sequencing data from TCGA database and found that ATF2 expression was significantly increased in the LUAD tissues compared with normal lung tissues (Fig. 1b). The result was further validated in paired LUAD and normal lung tissues in TCGA cohort (Fig. 1c). We also found that the expression of ATF2 in recurrent LUAD tissues was dramatically increased compared with primary LUAD tissues (Fig. 1d). Furthermore, protein expression analysis using Clinical Proteomic Tumor Analysis Consortium (CPTAC) data in ULCAN cancer database (http://ualcan.path.uab.edu/index.html) showed that ATF2 protein levels in LUAD tissues were higher than in the normal lung tissues (Fig. 1e). To validate the above results, we conducted qRT-PCR analysis with clinical LUAD specimens and lung cancer cell lines. QRT-PCR results showed that ATF2 expression in cancer tissues and cell lines was higher than in normal adjacent tissues and normal bronchial epithelial cell line, respectively (Fig. 1f, g). Although not statistically significant, disease-free survival (DFS) of patients in the ATF2 high-expression group was slightly shorter than in the ATF2 low-expression group (Fig. 1h). These results suggest that increased ATF2 expression is associated with LUAD progression.

### ATF2 promotes cell growth and invasion in LUAD cells

**Fig. 2:**
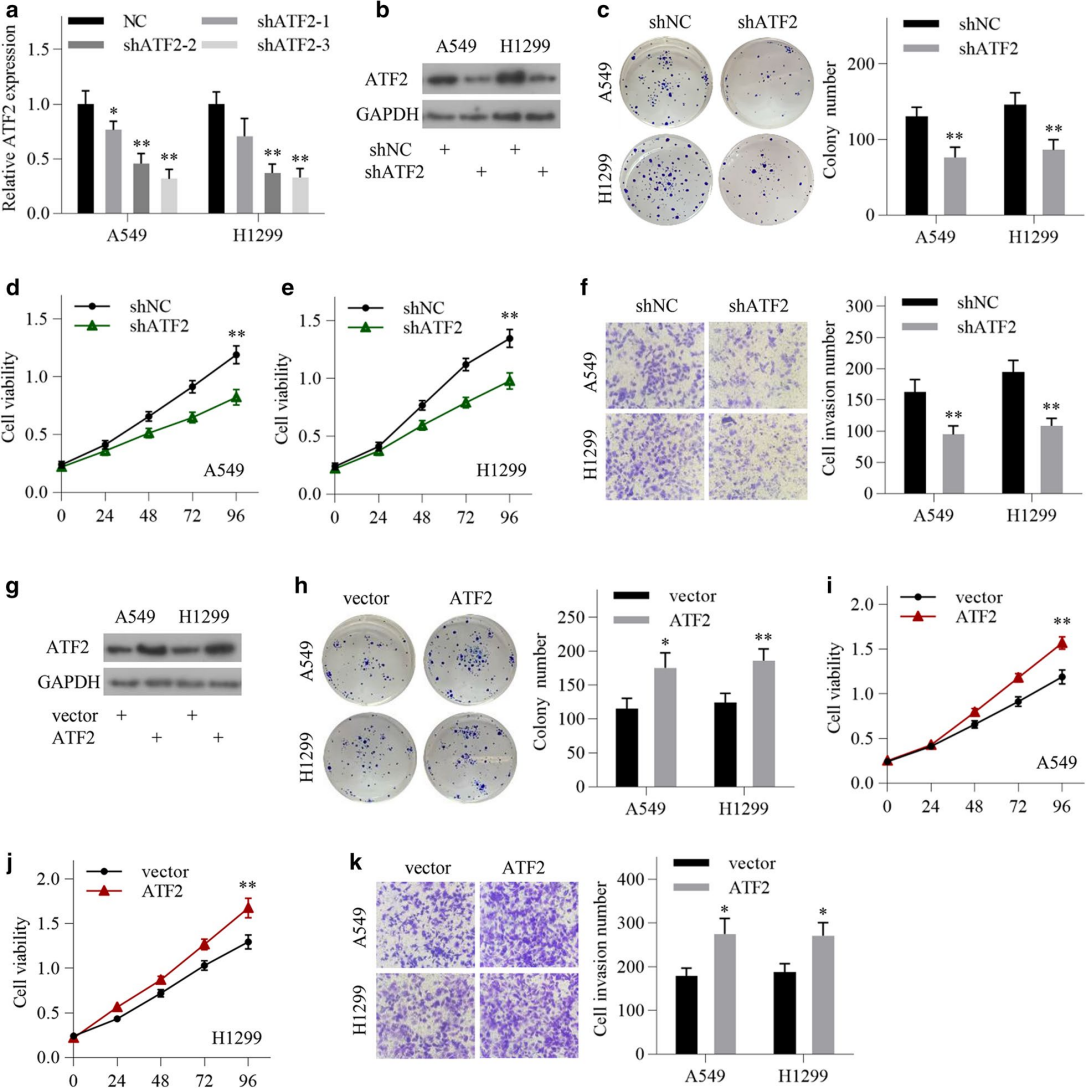
ATF2 promotes cell proliferation and invasion in LUAD cells. **a** The knockdown efficiency of three shRNA was detected by qRT-PCR in A549 and H1299 cells. **b** The knockdown efficiency of the shRNA for following experiments was validated by western blotting. **c** Colony formation assay was conducted following ATF2 knockdown in LUAD cells. **d**, **e** Cell viability was detected by CCK8 assay following ATF2 knockdown in A549 and H1299 cells. **f** Invasion ability of LUAD cells was determined by transwell invasion assay following ATF2 knockdown. **g** The expression of ATF2 protein was detected by western blotting after transfection with ATF2-overexpressing vector and empty vector in LUAD cells. **h** Colony formation assay was conducted following ATF2 overexpression in LUAD cells. **i**, **j** Cell viability was detected by CCK8 assay following ATF2 overexpression in A549 and H1299 cells. **k** Invasion ability of LUAD cells was detected by transwell invasion assay following ATF2 overexpression in LUAD cells. All data are shown as the mean ± SD of three independent experiments. ^*^P < 0.05, ^**^P < 0.01

To explore the potential role of ATF2 in LUAD progression, we first used shRNA to knock down the endogenous expression of ATF2 in LUAD cells. The shRNA that exhibited the highest interference efficiency was chosen for the following experiments (Fig. 2a). The interference efficiency was further validated by western blotting (Fig. 2b). Colony formation assay, CCK-8 assay and transwell invasion assay showed that ATF2 knockdown significantly inhibited cell proliferation and invasion in LUAD cells (Fig. 2c–f). Then we used an overexpressing plasmid to upregulate ATF2 levels in LUAD cells (Fig. 2g). The functional experiments showed that ATF2 overexpression significantly promoted cell proliferation and invasion in LUAD cells (Fig. 2h–k). To confirm whether the knockdown effects are specific, we constructed a mutant ATF2 plasmid that was insensitive to the shRNA used in the study (Additional file 3: Fig S1A). The add-back rescue experiment showed that the reduced colony formation ability induced by ATF2 knockdown were reversed by the mutant ATF2 plasmid (Additional file 3: Fig S1B). These results suggest that ATF2 promotes cell growth and invasion in LUAD cells.

### ATF2 activates NEAT1 transcription in LUAD cells

**Fig. 3:**
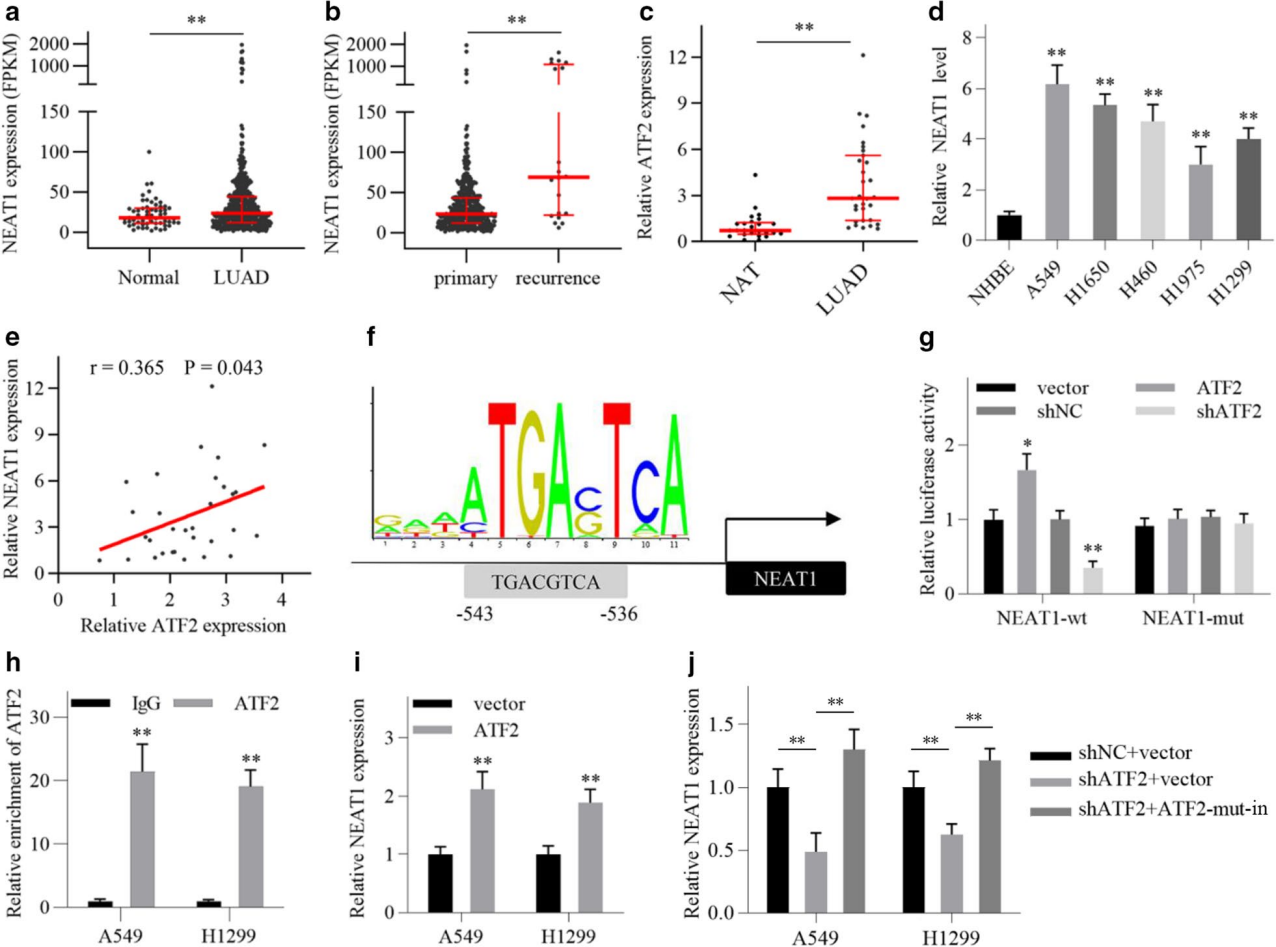
ATF2 activates NEAT1 transcription in LUAD cells. **a** NEAT1 expression in LUAD tissues and normal lung tissues from TCGA database. Normal, n = 59, LUAD, n = 533. **b** NEAT1 expression in primary and recurrent LUAD tissues from TCGA database. Primary, n = 513, Recurrence, n = 20. **c** NEAT1 expression in LUAD and normal lung tissues was detected by qRT-PCR. Normal adjacent lung tissues (NAT), n = 25, LUAD, n = 31. **d** NEAT1 expression in LUAD and normal bronchial epithelial cell lines was detected by qRT-PCR. **e** A positive correlation between NEAT1 and ATF2 expression in LUAD tissues was identified by qRT-PCR analysis. (0.2 ≤|r|< 0.4, low linear correlation). **f** Schematic showing the putative ATF2-binding site in the NEAT1 promoter region. **g** Luciferase activity assay was performed in A549 cells after transfection as indicated. **h** ChIP assay was performed for detecting ATF2 occupancy on the NEAT1 promoter region. **i**, **j** Relative expression of NEAT1 was detected by qRT-PCR in LUAD cells after transfection as indicated. ATF2-mut-in, the shRNA-insensitive mutant ATF2 plasmid. All data are shown as the mean ± SD of three independent experiments. ^*^P < 0.05, ^**^P < 0.01

Similar to the expression of ATF2, NEAT1 expression in LUAD tissues was significantly increased compared with normal lung tissues in TCGA cohort (Fig. 3a). The expression of NEAT1 in recurrent LUAD tissues was dramatically increased compared with primary LUAD tissues (Fig. 3b). In addition, qRT-PCR analysis showed that NEAT1 expression in LUAD tissues and cell lines was increased compared with normal lung cancer tissues and normal bronchial epithelial cell line, respectively (Fig. 3c, d). Remarkably, a positive correlation between NEAT1 and ATF2 expression in LUAD tissues was identified by qRT-PCR analysis, indicating a potential interplay between NEAT1 and ATF2 in LUAD (Fig. 3e).

Given that ATF2 is a transcription factor, we wondered whether ATF2 could activate NEAT1 transcription in LUAD cells. Then we searched for putative ATF2-binding sites in the NEAT1 promoter by using the JASPAR (http://jaspardev.genereg.net/) and PROMO databases (http://alggen.lsi.upc.es/cgi-bin/promo_v3/promo/promoinit.cgi?dirDB=TF_8.3), and one binding site was predicted by both the databases (Fig. 3f). To investigate whether this site was functional, we constructed a wild-type and a mutated luciferase reporter vector containing the wild or mutated predicted binding site, respectively. The luciferase activity assay showed that ATF2 knockdown significantly reduced the luciferase activity of wild-type vector, while ATF2 overexpression promoted it. When the biding site was mutated, the luciferase activity was unaffected (Fig. 3g). Furthermore, ChIP analysis showed that ATF2 was significantly enriched in the NEAT1 promoter in LUAD cells (Fig. 3h). QRT-PCR analysis showed that NEAT1 expression was significantly increased following ATF2 overexpression in LUAD cells, while decreased following ATF2 knockdown (Fig. 3i, j). Moreover, the reduced NEAT1 levels induced by ATF2 knockdown were reversed by the shRNA-insensitive mutant ATF2 plasmid (Fig. 3j). These results suggest that ATF2 directly activates NEAT1 transcription by binding to the NEAT1 promoter.

### ATF2/NEAT1 axis is involved in the progression of LUAD

**Fig. 4:**
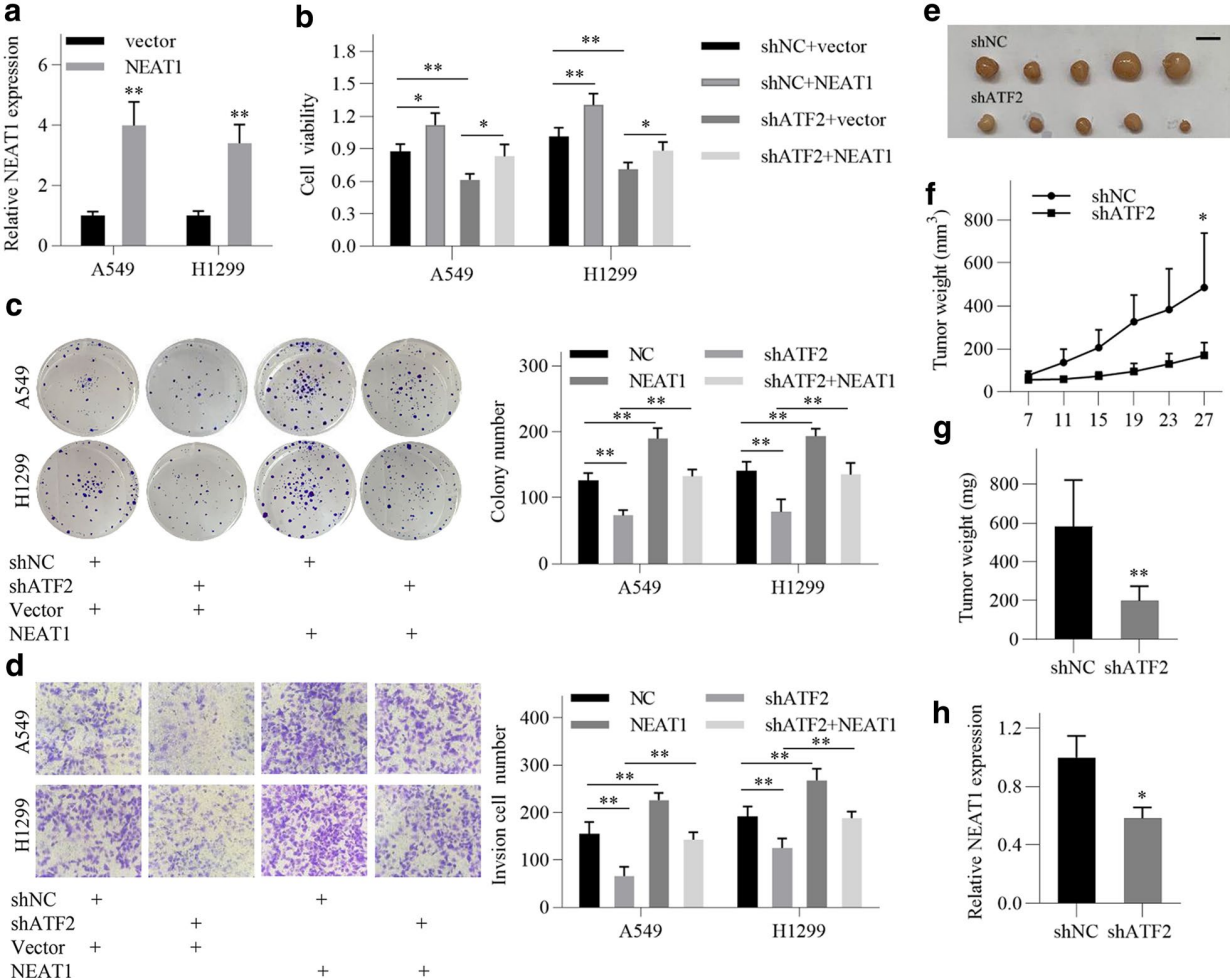
ATF2/NEAT1 axis is involved in the progression of LUAD. **a** Relative expression of NEAT1 was detected by qRT-PCR after transfection with NEAT1-overexpressing vector and empty vector in LUAD cells. **b** Cell viability of LUAD cells was detected by CCK8 assay after transfection as indicated. **c** Colony formation ability of LUAD cells was detected after transfection as indicated. **d** Invasion ability of LUAD cells was detected by transwell invasion assay after transfection as indicated. **e** A photograph of the tumors collected from nude mice in the shATF2 and shNC group (n = 5). Scale bar = 1 cm. **f** The tumor volumes were detected at the indicated time. **g** The tumor weights were measured at the termination of the experiment. **h** The expression of NEAT1 in tumor tissues was detected by qRT-PCR. All of the data are shown as the means ± SD. ^*^P < 0.05; ^**^P < 0.01

To investigate the role of ATF2/NEAT1 axis in LUAD, ATF2 shRNA and pcDNA-NEAT1 vector were co-transfected into the LUAD cells. The transfection effect of pcDNA-NEAT1 vector was confirmed by qRT-PCR (Fig. 4a). Functional experiments results showed that NEAT1 overexpression promoted cell proliferation and invasion in LUAD cells (Fig. 4b–d). Moreover, the reduced cell proliferation and invasion induced by ATF2 knockdown were reversed by NEAT1 overexpression (Fig. 4b–d). These findings indicate that ATF2 promotes the proliferation and invasion of LUAD cells, at least, partly via upregulating NEAT1 in vitro.

A xenograft tumor model was established to validate the role of ATF2/NEAT1 axis in LUAD progression in vivo. We found that the proliferative activity of the tumors was lowered in the shATF2 group than in the shNC group (Fig. 4e–g). In addition, qRT-PCR analysis showed that the expression of NEAT1 was reduced in the shATF2 group compared with the shNC group (Fig. 4h). These results suggest that ATF2/NEAT1 axis is involved in LUAD progression in vivo.

### NEAT1 promotes ATF2 expression and forms a positive feedback loop

**Fig. 5:**
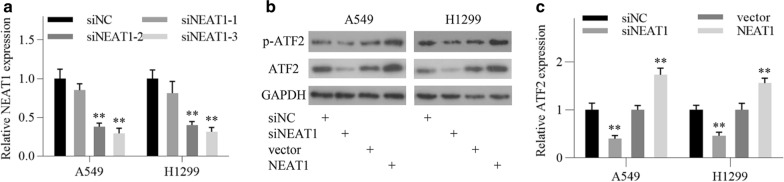
NEAT1 positively regulates ATF2 expression in LUAD cells. **a** The knockdown efficiency of three siRNA for NEAT1was detected by qRT-PCR in A549 and H1299 cells. **b** ATF2 and p-ATF2 levels were detected by western blotting after transfection as indicated. **c** Relative expression levels of ATF2 mRNA were detected by qRT-PCR after transfection as indicated. All data are shown as the mean ± SD of three independent experiments. ^*^P < 0.05, ^**^P < 0.01

Previous studies have proved that NEAT1 is involved in the activation of MAPK signaling pathway in a variety of pathological processes [22, 26, 27], so we wondered whether NEAT1 could regulate ATF2 expression in LUAD cells. To validate this hypothesis, we knocked down the expression of NEAT1 in LUAD cells by using three siRNA, and the one with the highest interference efficiency was chosen for the following experiments (Fig. 5a). Western blot analysis showed that NEAT1 knockdown significantly inhibited ATF2 and p-ATF2 levels in LUAD cells, while NEAT1 overexpression achieved the opposite effect (Fig. 5b). Furthermore, qRT-PCR analysis revealed that the mRNA expression of ATF2 was positively regulated by NEAT1 (Fig. 5c). These results suggest that NEAT1 promotes ATF2 expression, creating a positive feedback loop in LUAD.

### NEAT1 is a direct target of miR-26a in LUAD cells

**Fig. 6:**
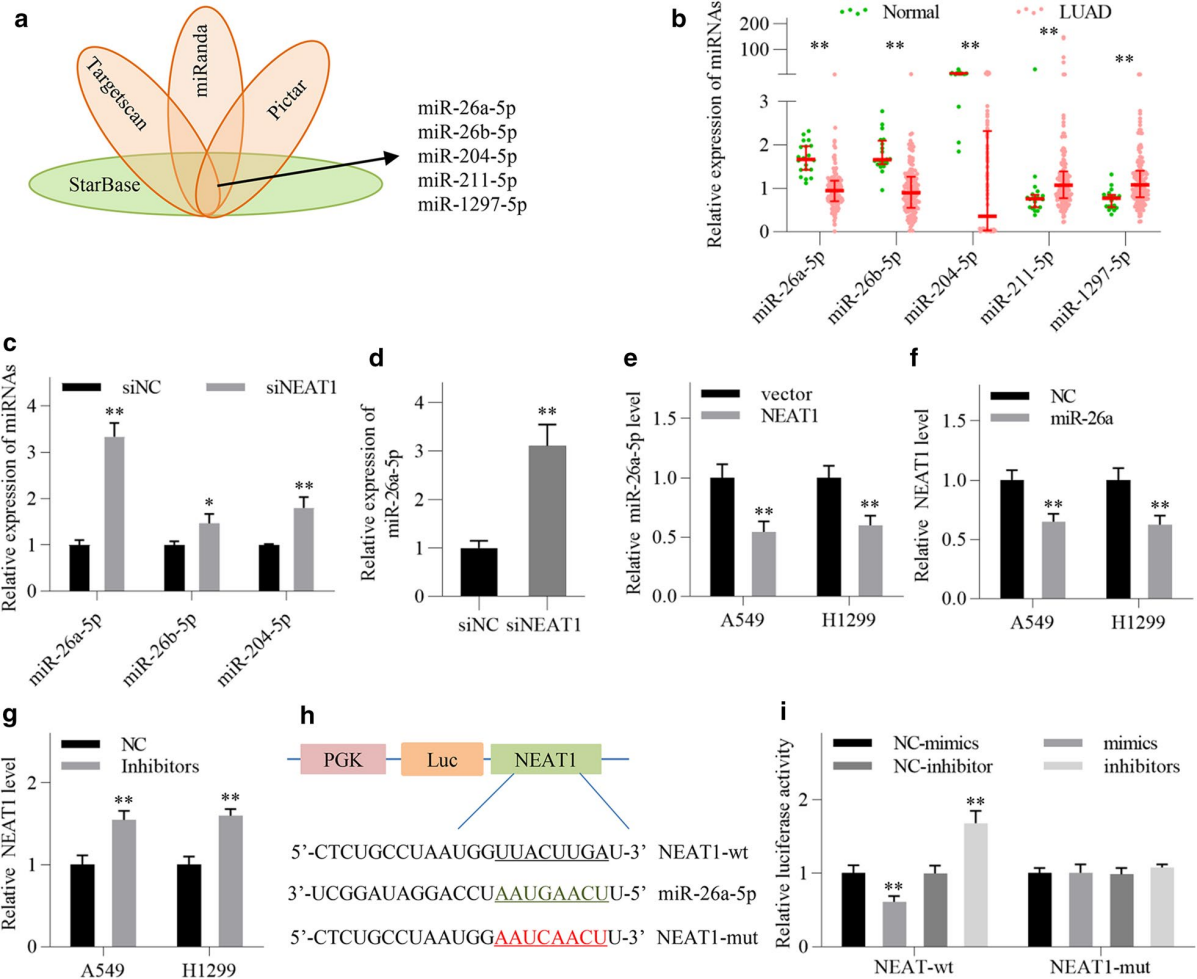
NEAT1 is a direct target of miR-26a in LUAD cells. **a** Four online tools (miRanda, Targetscan, Pictar and StarBase) were used to predict the miRNAs that target both NEAT1 and ATF2 3′UTR. **b** The expression of the five predicted miRNAs in LUAD tissues and normal lung tissues was analyzed using GSE48414 dataset. Normal, *n* = 20, LUAD, *n* = 154. **c** MiR-26a-5p, miR-26b-5p and miR-204-5p levels in A549 cells were detected by qRT-PCR following NEAT1 knockdown. **d** MiR-26a-5p expression in H1299 cells was detected by qRT-PCR following NEAT1 knockdown. **e** miR-26a-5p expression in LUAD cells was detected by qRT-PCR following NEAT1 overexpression. **f**, **g** NEAT1 expression was detected by qRT-PCR in LUAD cells after transfection with miR-26a-5p mimics and inhibitors, respectively. **h** Schematic representation of the putative binding site between miR-26a-5p and NEAT1. **i** Luciferase activity assay was performed in A549 cells after transfection as indicated. All data are shown as the mean ± SD of three independent experiments. ^*^P < 0.05, ^**^P < 0.01

We next investigated the molecular mechanism by which NEAT1 promoted ATF2 transcription. Previous studies have suggested that ceRNA is the main mechanism of NEAT1 through which regulates target genes [18]. So we searched for the putative miRNAs targeting both NEAT1 transcript and ATF2 3′UTR by using StarBase, Targetscan, miRanda and Pictar online databases. A total of five miRNAs were screened out, namely, miR-26a-5p, miR-26b-5p, miR-204-5p, miR-211-5p, miR-1297-5p (Fig. 6a). Then we investigated the expression of the five miRNAs in LUAD tissues by employing a GEO dataset (GSE48414). The results showed that miR-26a-5p, miR-26b-5p and miR-204-5p were significantly reduced in LUAD tissues compared with normal lung tissues (Fig. 6b). Subsequently, we found that the three miRNAs levels were all increased following NEAT1 knockdown in A549 cells, and miR-26a-5p exhibited a more significant increase than the others (Fig. 6c). The expression of miR-26a-5p in H1299 cells was also significantly elevated following NEAT1 knockdown (Fig. 6d). Conversely, miR-26a-5p levels were significantly reduced by NEAT1 overexpression in LUAD cells (Fig. 6e). For these reasons, we chose miR-26a-5p as main research molecule for the following experiments. What’s more, the expression of NEAT1 following miR-26a-5p knockdown or overexpression was detected by qRT-PCR. The results showed that NEAT1 levels were reduced by miR-26a-5p overexpression, while increased by miR-26a-5p knockdown (Fig. 6f, g).

We then constructed two luciferase reporter vectors with wild-type or mutant NEAT1 fragments containing the putative miR-26a-5p binding site (Fig. 6h). Luciferase activity assay showed that miR-26a-5p overexpression significantly reduced the luciferase activity of wild-type NEAT1 vector, while miR-26a-5p knockdown increased it (Fig. 6i). However, the luciferase activity of mutated NEAT1 vector was not affected following miR-26a-5p mimics or inhibitors transfection (Fig. 6i). The above results suggest that NEAT1 is a direct target of miR-26a in LUAD cells.

### *NEAT1 positively regulates ATF2 *via* sponging miR-26a-5p in LUAD cells*

**Fig. 7:**
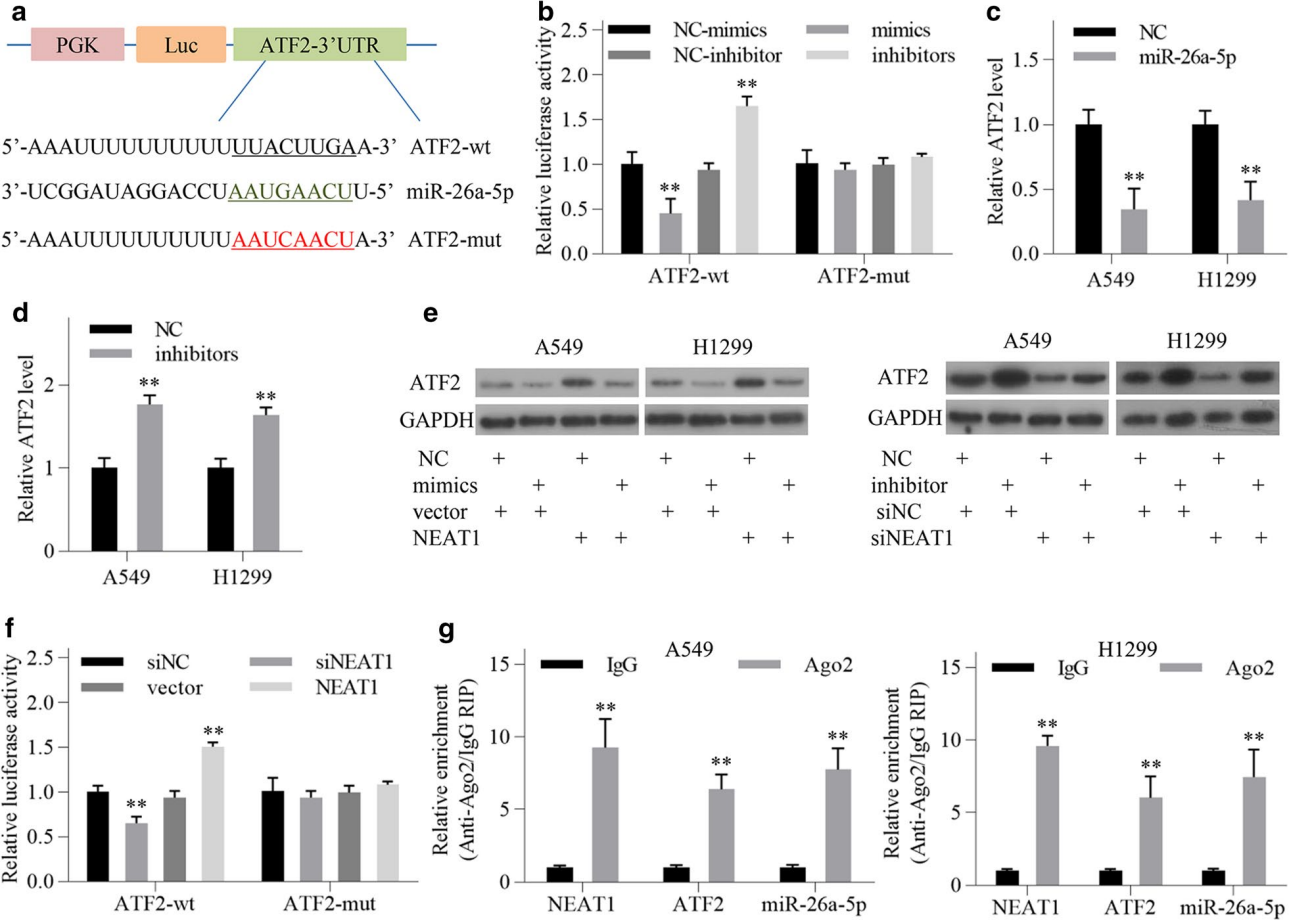
NEAT1 positively regulates ATF2 expression via sponging miR-26a-5p in LUAD cells. **a** Schematic representation of the putative binding site between miR-26a-5p and ATF2. **b** Luciferase activity assay was performed in A549 cells after transfection as indicated. **c**, **d** Relative expression levels of ATF2 mRNA in LUAD cells were detected by qRT-PCR after transfection with miR-26a-5p mimics and inhibitors, respectively. **e** ATF2 protein levels were detected by western blotting after transfection as indicated. **f** Luciferase activity assay was performed in A549 cells after transfection as indicated. **g** RNA immunoprecipitation assay was performed to determine the amount of NEAT1, ATF2 and miR-26a-5p pulled down by anti-Ago2 antibody in A549 and H1299 cells. All data are shown as the mean ± SD of three independent experiments. ^*^P < 0.05, ^**^P < 0.01

To investigate whether ATF2 is a direct target of miR-26a-5p, we constructed two luciferase reporter vectors containing wild-type or mutant ATF2 3′UTR fragments harboring the miR-26a-5p binding site, respectively (Fig. 7a). Luciferase activity assay showed that miR-26a-5p overexpression significantly reduced the luciferase activity of wild-type ATF2 vector, whereas miR-26a-5p knockdown enhanced the luciferase activity (Fig. 7b). However, the luciferase activity of mutated ATF2 vector was not affected (Fig. 7b). QRT-PCR analysis showed that ATF2 mRNA levels were reduced following miR-26a-5p mimics transfection in LUAD cells, while elevated following miR-26a-5p inhibitors transfection (Fig. 7c, d). Western blot analysis showed that ATF2 protein levels were negatively regulated by miR-26a-5p (Fig. 7e). The above results suggest that ATF2 is a direct target of miR-26a-5p in LUAD cells.

Furthermore, we found that miR-26a-5p overexpression or knockdown attenuated the effect of NEAT1-overexpressing vector or NEAT1 siRNA on ATF2 protein expression, respectively (Fig. 7e). Luciferase activity assay showed that NEAT1 knockdown reduced the luciferase activity of wild-type ATF2 vector, whereas NEAT1 overexpression enhanced it (Fig. 7F). Argonaute 2 (Ago2) is the central component of the RNA-induced silencing complex (RISC) that is necessary for miRNA-mediated gene silencing [28]. RIP assay indicated that NEAT1, miR-26a-5p, and ATF2 were all abundant in anti-Ago2 group compared with anti-IgG group, suggesting the coexistence of them in RISC in LUAD cells (Fig. 7g). These results suggest that NEAT1 positively regulates ATF2 expression via sponging miR-26a in LUAD cells.

## Discussion

Previous studies have demonstrated that elevated ATF2 expression is associated with cell proliferation, cisplatin resistance and poor prognosis in NSCLC [13, 14, 29]. In this study, we confirmed that ATF2 levels were increased in LUAD tissues compared with normal lung tissues. Furthermore, we demonstrated that ATF2 bound to the NEAT1 promoter and activated NEAT1 gene transcription. Functional experiments showed that ATF2 promoted cell proliferation and invasion in LUAD cells via upregulating NEAT1 expression. In turn, NEAT1 promoted ATF2 expression via sponging miR-26a-5p, and formed a positive feedback loop to promote the progression of LUAD.

Studies have shown that ATF2 acts as an oncogene via promoting target genes transcription in many cancers [5]. Moreover, ATF2 is reported to be involved in the crosstalk between paraspeckles and mitochondrial signaling [30]. In this study, we found that ATF2 levels in LUAD tissues were positively correlated with NEAT1 levels. Subsequently, we demonstrated that ATF2 promoted NEAT1 transcription via binding to the NEAT1 promoter in LUAD cells. In hepatocellular carcinoma, BCLAF1 promotes cell proliferation, invasion and 5-Fluorouracil resistance though targeting NEAT1 [31]. In lung cancer cells, NEAT1 and MALAT1 have been proved to be downstream effectors of Oct4-induced lung cancer proliferation, migration and invasion [21]. Given that NEAT1 was involved in lung cancer tumorigenesis and metastasis, we next investigated whether ATF2 exerted its oncogenic role through upregulating NEAT1. Rescue experiments results showed that NEAT1 overexpression abolished the effects of ATF2 knockdown on cell proliferation and invasion in LUAD cells, indicating that NEAT1 was a downstream effector of ATF2 in LUAD.

NEAT1 is involved in TLR4-mediated inflammatory process mainly through activating the late MAPK pathways, including the phosphorylation activation of JNK, p38 and ERK1 [27]. Moreover, NEAT1 increases MAPK pathway activity via sponging let-7a in cancer cells, resulting in increased phosphorylation of ERK1/2 and p38 [22, 26]. Coincidentally, ATF2 is activated by JNK, p38 or ERK1 via Thr69 and Thr71-phosphorylation [4]. So we wondered whether the activity of ATF2 was regulated by NEAT1 in LUAD. In this study, we found that NEAT1 positively regulated ATF2 and p-ATF2 levels, indicating a positive feedback loop between ATF2 and NEAT1 in LUAD cells. The positive feedback loops could amplify the oncogenic effects of interaction molecules [32], such as ATF2 and NEAT1 in this study, and thus more significantly promote the progression of LUAD. Moreover, we found that ATF2 mRNA levels in LUAD cells were significantly reduced by NEAT1 knockdown, whereas elevated by NEAT1 overexpression, suggesting a novel transcriptional regulation mechanism of ATF2. Subsequently, we explored the mechanism of NEAT1-induced ATF2 expression.

NEAT1 has been reported to regulate gene expression by a variety of mechanisms. For example, NEAT1 functions as a scaffold molecule of EZH2 to repress the expression of Wnt/β-catenin pathway negative regulators, such as AXIN2, GSK3β and ICAT, in an H3K27me3-dependent manner [33]. NEAT1 represses the expression of E-cadherin and miR-129 via inducing CpG island DNA hypermethylation in the promoter regions [34, 35]. However, NEAT1 positively regulates downstream oncogenes expression mainly via acting as a ceRNA in cancers [18]. Moreover, it has been demonstrated that NEAT1 is not only enriched in the nuclear but also found in the cytoplasm in cancer cells [36, 37]. As a result, we supposed that NEAT1 probably acted as a ceRNA to positively regulated ATF2 expression via sponging some miRNAs. Then we screened out a few putative miRNAs targeting both NEAT1 and ATF2 3′UTR using online prediction databases. Subsequently, we demonstrated that NEAT1 positively regulated ATF2 expression via sponging miR-26a-5p in LUAD cells.

## Conclusion

In summary, we demonstrated that ATF2 is elevated in LUAD and plays an important role in cell proliferation and invasion in LUAD cells. Mechanistically, ATF2 and NEAT1 form a positive feedback loop mediated by miR-26a-5p and coordinately promote LUAD progression. Based on these findings, blocking the positive feedback loop involving ATF2 and NEAT1 might represent a new strategy for the treatment of LUAD.

## Supplementary Information

**Additional file 1**: **Table S1**. The oligonucleotides used in this study.

**Additional file 2**: **Table S2**. The primers used in this study.

**Additional file 3: Figure S1.** The add-back rescue experiment. (A) The shRNA-insensitive mutant ATF2 plasmid (ATF2-mut-in) was validated by qRT-PCR. (B) Colony formation ability of ATF2 knockdown LUAD cells was detected after transfection as indicated. ns, not significant. ^**^P < 0.01.

## Data Availability

All data generated or analyzed during this study are included in this published article and its additional files.

## Abbreviations

ATF2: Activating transcription factor 2
AP-1: Activator protein 1
LUAD: Lung adenocarcinoma
NSCLC: Non-small cell lung cancer
LncRNA: Long non-coding RNA
NEAT1: Nuclear Enriched Abundant Transcript 1
ceRNA: Competitive endogenous RNA
DMEM: Dulbecco minimal essential medium
FBS: Fetal bovine serum
siRNA: Small interfering RNA
shRNA: Short hairpin RNA
QRT-PCR: Quantitative Realtime PCR
ChIP: Chromatin immunoprecipitation
RIP: RNA immunoprecipitation
TCGA: The Cancer Genome Atlas
GEO: The Gene Expression Omnibus
