# Supplementary material for: The interplay between ATF2 and NEAT1 contributes to lung adenocarcinoma progression
Source: Cancer Cell Int. 2020 Dec 9;20:594. doi: 10.1186/s12935-020-01697-8 (PMC7727147; doi:10.1186/s12935-020-01697-8)
Supplement: Supplementary file 1 — Additional file 1: Table S1. The oligonucleotides used in this study. [file 12935_2020_1697_MOESM1_ESM.docx]

Table S1. The oligonucleotides used in this study.

| Name | Target sequence (5’-3’) |
| --- | --- |
| ATF2-shRNA1 | GGAAGTACCATTGGCACAA |
| ATF2-shRNA2 | ATCATTACAGGTTCCCAAT |
| ATF2-shRNA3 | GGCTATCATACTGCTGATA |
| NEAT1-siRNA1 | CTGGCATGCTGAAGAAGAA |
| NEAT1-siRNA2 | GGAATCTGTGTTTGTGTGA |
| NEAT1-siRNA3 | GCACATGTTTGAACTTCAA |
| miR-26a-5p mimics-sense | UUCAAGUAAUCCAGGAUAGGCU |
| miR-26a-5p mimics-antisense | CCUAUCCUGGAUUACUUGAAUU |
| miR-26a-5p inhibitors | AGCCUAUCCUGGAUUACUUGAA |
