# Supplementary material for: The interplay between ATF2 and NEAT1 contributes to lung adenocarcinoma progression
Source: Cancer Cell Int. 2020 Dec 9;20:594. doi: 10.1186/s12935-020-01697-8 (PMC7727147; doi:10.1186/s12935-020-01697-8)
Supplement: Supplementary file 2 — Additional file 2: Table S2. The primers used in this study. [file 12935_2020_1697_MOESM2_ESM.docx]

Table S2. The primers used in this study.

| Name | Sequence (5’-3’) |
| --- | --- |
| ATF2-F | AGATTTATTAATTTTTCTGTGCTCAA |
| ATF2-R | ACACCCCCATTTATTAAAACACC |
| NEAT1-F | CTTCCTCCCTTTAACTTATCCATTCAC |
| NEAT1-R | CTCTTCCTCCACCATTACCAACAATAC |
| ACTB-F | CCTTCTACAATGAGCTGCGT |
| ACTB-R | CCTGGATAGCAACGTACATG |
| miR-26a-5p-F | CTGTCAACGATACGCTAC |
| miR-26a-5p-R | GTAATCCAGGATAGGCTG |
| miR-26b-5p-F | CGCCCTGTTCTCCATTACTT |
| miR-26b-5p-R | CCAGTGCAGGGTCCGAGGT |
| miR-204-5p-F | CGGCGTTTGTCATCCTATG |
| miR-204-5p-R | GTGCAGGGTCCGAGGT |
| U6-F | CTCGCTTCGGCAGCACA |
| U6-R | AACGCTTCACGAATTTGCGT |
| NEAT1-ChIP-F | ACGGCCTCTTCCCACTTAAT |
| NEAT1-ChIP-R | AGGCATCGTGGTTTTGACTC |
