# Supplementary figures and images for: The interplay between ATF2 and NEAT1 contributes to lung adenocarcinoma progression
Source: Cancer Cell Int. 2020 Dec 9;20:594. doi: 10.1186/s12935-020-01697-8 (PMC7727147; doi:10.1186/s12935-020-01697-8)

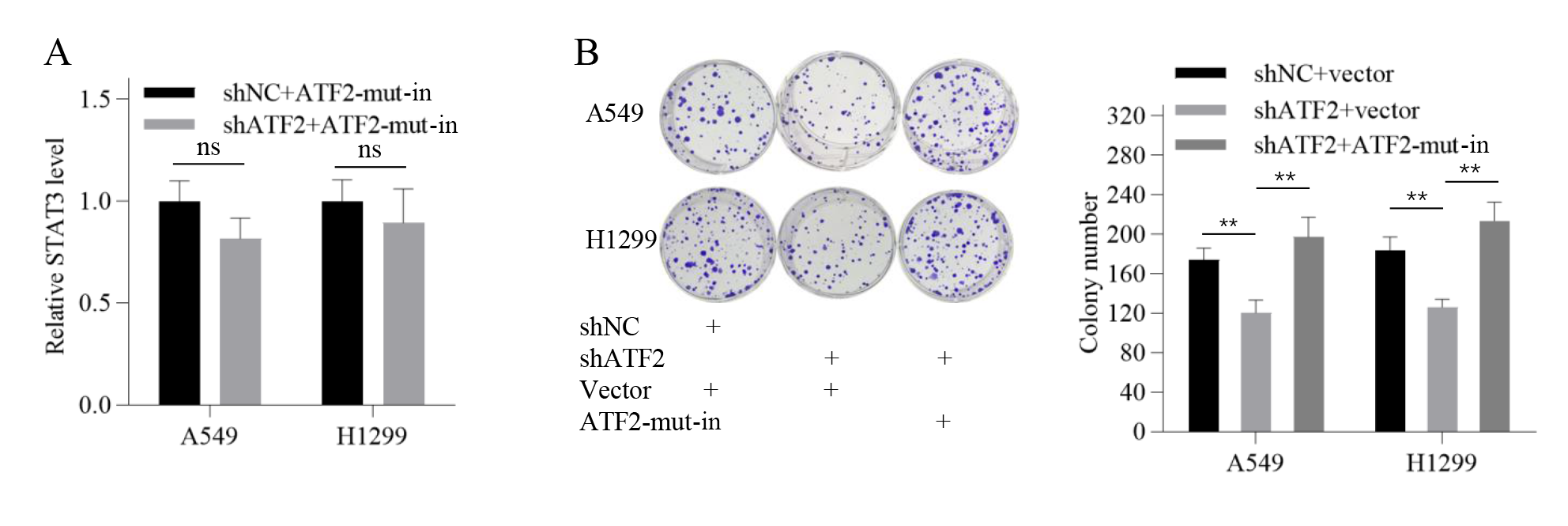

Supplement: Supplementary file 3 — Additional file 3: Figure S1. The add-back rescue experiment. (A) The shRNA-insensitive mutant ATF2 plasmid (ATF2-mut-in) was validated by qRT-PCR. (B) Colony formation ability of ATF2 knockdown LUAD cells was detected after transfection as indicated. ns, not significant. **P < 0.01. [file 12935_2020_1697_MOESM3_ESM.tif]
